# Effect of BMI and Its Optimal Cut-Off Value in Identifying Hypertension in Uyghur and Han Chinese: A Biethnic Study from the China National Health Survey (CNHS)

**DOI:** 10.1155/2018/1508083

**Published:** 2018-12-11

**Authors:** Huijing He, Lize Pa, Li Pan, Adili Simayi, Hebuli Mu, Yashengjiang Abudurexiti, Ning Tao, Guangliang Shan

**Affiliations:** ^1^Department of Epidemiology and Statistics, Institute of Basic Medical Sciences, Chinese Academy of Medical Sciences and Department of Epidemiology and Statistics, School of Basic Medicine, Peking Union Medical College, Beijing 100005, China; ^2^Xinjiang Uygur Autonomous Region Center for Disease Control and Prevention, Urumqi 830000, China; ^3^Department of Epidemiology and Statistics, College of Public Health, Xinjiang Medical University, Urumqi 830054, China

## Abstract

**Objective:**

The effect of adiposity on hypertension among Uyghur Chinese is not clear. This study aimed to compare the effect of BMI and its optimal cut-off value in identifying hypertension in Uyghur and Han adults in China.

**Methods:**

By using a multistage stratified sampling method, 3072 Uyghur and 3195 Han adults underwent questionnaire interview, physical examination, and biochemical tests. Age- and sex-standardized prevalence of hypertension was calculated. Adjusted odds ratios for adiposity associated with hypertension were estimated. ROC analyses were used for assessing the ethnic and sex specific optimal BMI cut-off values in identifying hypertension.

**Results:**

Both in Uyghur and Han, increased BMI was consistent with the elevated systolic and diastolic blood pressure. Although more Uyghur were overweight/obese, their standardized prevalence of hypertension (17.87%) was lower than that of Han (20.28%). Han adults had 1.42 times odds than Uyghur of hypertension. The adjusted ORs of overweight and obesity were 2.67 and 6.04 in Uyghur and 2.74 and 7.58 in Han. In male, the optimal cut-off values of BMI identifying hypertension in Uyghur and Han were 24.6 kg/m^2^ and 24.9 kg/m^2^ , respectively, but the correspond values in Uyghur and Han females were 27.2 kg/m^2^ and 25.0 kg/m^2^.

**Conclusions:**

Adiposity had strong effect on hypertension, but this effect was less strong in Uyghur female than in Han female.

## 1. Introduction

World widely, hypertension is a major public health problem and is considered the greatest attributable risk factors for death [1]. Although blood pressure control is a national public health priority in China [2], the prevalence of hypertension in Chinese adults aged 35-75 was 44.7% in 2017 [3]. Identifying modifiable risk factors of hypertension is of great concern. Excess adiposity is a well-established risk factor for major chronic diseases [4], and it is generally accepted that being overweight or obese increases the risk of the development of hypertension [5–7].

Ethnic disparities in obesity could result in concurrent differential prevalence of obesity-related diseases including hypertension [8], and this varied effect has been observed previously [9, 10]. China has a diversity of ethnic groups having different cultures and heterogeneity of socioeconomic levels as well as genetic backgrounds. According to the 2010 census data [11], Uyghur Chinese ranked the forth population size (approximately 10.1 million) of the minority ethnicities in China, and the majority of them live in the Xinjiang Uyghur Autonomous Region located in the northwest China. Previous studies have explored the disparities between Uyghur and Han on prevalence of several chronic diseases such as type 2 diabetes, dyslipidemia, and hypertension [11–14]. Comparisons on hypertension prevalence have inconsistent results. For example, Gu's study revealed a higher prevalence in Uyghur adults than Han [13], in contrast with Tao's study with opposite finding [14]. Moreover, the effect of adiposity on hypertension in Uyghur is unclear.

Therefore, in this cross-sectional study, we used data from the China National Health Survey (CNHS) to investigate the role of body mass index (BMI) as risk factor of hypertension in Uyghur and Han adults in China. We hypothesize that different genetic background, socioeconomic status, and life style between Uyghur and Han could probably lead to disparities in hypertension prevalence as well as modify the effect of adiposity as risk factor of hypertension.

## 2. Materials and Methods

### 2.1. Study Population

Details of the CNHS study design and methods have been described previously [15, 16]. The CNHS is an ongoing population-based cross-sectional study and Xinjiang Uyghur Autonomous Region is one of the study sites, where 3072 Uyghur and 3195 Han adults aged 20-80 were enrolled in 2013. The ethnicity of Uyghur or Han was defined strictly as participants having concordant officially recorded ethnic identity with both of their parents.

### 2.2. Measurements

A comprehensive questionnaire including information on demographic and socioeconomic characteristics, personal and family medical history, and life style risk factors was administered by trained interviewers. Anthropometric measurements including height, weight, and body composition were taken using calibrated instruments with standard protocols, with participants wearing light clothing and bared feet. Weight and body composition were measured using a body composition analyzer (TANITA BC-420, Japan). Standing height was obtained to decimal level precision by a stadiometer. Body mass index was calculated as weight in kilograms divided by the square of height in meters (kg/m^2^). For each participant, systolic blood pressure (SBP) and diastolic blood pressure (DBP) were measured three times after at least five minutes rest in a seated position, using a digital sphygmomanometer (Omron HEM-907, Japan). A 9-ml fasting blood sample with at least 8 hours fasting overnight was collected. Fasting plasma glucose (FPG, mmol/L), serum-lipid including triglyceride (TG, mmol/L), total cholesterol (TC, mmol/L), high-density lipoprotein cholesterol (HDL-C, mmol/L), and low-density lipoprotein cholesterol (LDL-C, mmol/L) were tested in the General Hospital of Chinese People's Liberation Army. Ethic approval was obtained from the Bioethical Committee of Institute of Basic Medical Sciences, Chinese Academy of Medical Sciences. All participants provided written informed consent before the questionnaire interview.

### 2.3. Variables

Excess adiposity was measured by BMI that was separated into three categories based on World Health Organization (WHO) classification [17]: under/normal weight (BMI <25 kg/m^2^), overweight (25 kg/m^2^ ≤BMI <30 kg/m^2^), and obesity (BMI ≥30 kg/m^2^). Hypertension was defined as individual who was measured with an average SBP ≥ 140mmHg and/or an average DBP ≥ 90mmHg, or self-reported hypertension history [15, 18]. The classification and definition of other variables, such as smoking, alcohol drinking, physical activity [19], diabetes, and dyslipidemia, were described in our previous publications [12, 15].

### 2.4. Statistical Analysis

In this study, we restricted analytic sample to participants with complete information on major risk factors (i.e., age, sex, ethnicity, height, weight, history of hypertension, and measurements of blood pressure). This analysis included 2928 Uyghur and 3097 Han adults. 242 participants were excluded because of missing value on major risk factors.

Summary results are presented as mean (standard deviation, SD) for continuous data and number (percentage, %) for categorical data. Since underweight subjects accounted only for 3.52% and 3.10% in Uyghur and Han, they were integrated into one category into analysis.

Scatter plots were drawn to identify the linear relationship between covariates and blood pressure. General linear regression models (GLMs) were used to perform two-way analysis of covariates (ANCOVA). For the comparison of hypertension prevalence, age- and sex-standardized prevalence was calculated by using the population census data of China in 2010. Multivariable logistic regression models were used to examine the association between excess adiposity and hypertension. Ethnic and sex specific receiver operating characteristic (ROC) curves were applied to evaluate the overall performance of BMI cut-off values in detecting excess risk for Uyghur and Han adults. The areas under the ROC curve (AUC) were calculated by logistic regression model and were used as a measure of accuracy of BMI in screening the presence of hypertension risk factors. The Youden index was calculated (sensitivity + specificity -1), and the maximum of it was considered as the optimal cut-off value for BMI.

We further did several sensitivity analyses to ascertain the effects of BMI on hypertension among participants (1) who were newly diagnosed with hypertension in the survey; (2) who were not diagnosed with diabetes to avoid the possible influence on BMI by lifestyle change. We also perform the analysis using body fat percentage as a replacement of BMI to compare indexes of body composition on the association with hypertension.

Statistical analysis was performed using SAS 9.4 (SAS Institute Inc. Cary, NC, USA).

## 3. Results

### 3.1. Demographic and Clinical Characteristics in Uyghur and Han Participants

2928 Uyghur (1028 males and 1900 females) and 3097 Han adults (1255 males and 1842 females) aged 20-80 years were included for analysis. Both in Uyghur and Han, female participants accounted more in proportion than male, with 64.9% in Uyghur and 59.5% in Han, respectively. Uyghur and Han had significant difference in educational attainment, residential areas, cigarette smoking, alcohol drinking, and physical activity (P<0.001). In the Uyghur participants, male had higher proportion on older age, higher educational attainment, urban resident, overweight/obesity, ever-smoking, alcohol consumption, moderate or active physical activity, and hypertension. This sex difference was also seen in Han group except for residential areas (P=0.355). The demographic and clinical characteristics across categories of ethnicity and sex appeared in Table 1.

### 3.2. The Relationship between BMI and Blood Pressure in Uyghur and Han Participants

In Uyghur male, the mean of SBP increased from 118.30 mmHg in the under/normal weight group to 131.81 mmHg in the obesity group (P<0.001). Likewise, DBP increased from 69.97 mmHg in the under/normal weight group to 81.13 mmHg in the obesity group (P<0.001). The same trend of increasing SBP and DBP with elevated BMI categories was also observed in Uyghur female and Han male and female (all P <0.001, supplementary S-table 1).

The means of SBP for Uyghur and Han were 119.25 mmHg and 120.08 mmHg. DBP in the two ethnic groups was 72.74 mmHg and 73.92 mmHg. After adjusting for age, sex, BMI, and alcohol consumption, Han adults had higher SBP (P=0.039) and DBP (P<0.001) level than Uyghur. But after the stratified analysis by sex, no statistical difference on SBP was found, but there was still higher DBP level in Han than in Uyghur (P<0.001) in both sexes.

### 3.3. The Prevalence of Hypertension and the Association with BMI

**Figure 1:**
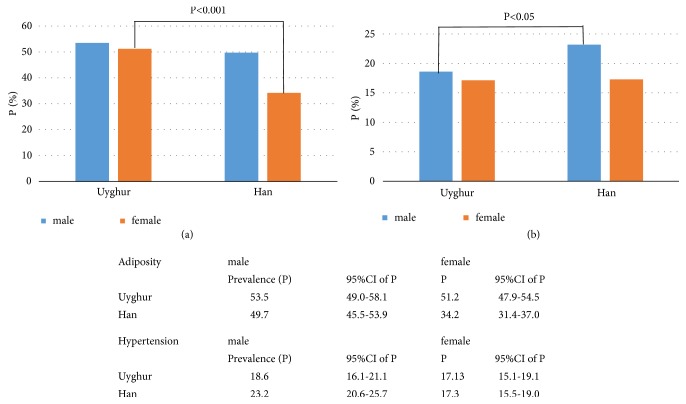
The sex specific prevalence of overweight/obesity and hypertension in Uyghur and Han adults. (a) Adiposity (defined as overweight or obesity); (b) hypertension; Pc: crude prevalence; Ps: standardized prevalence.

The age- and sex-standardized hypertension prevalence was 17.87% (95% CI: 16.26% to 19.48%) in Uyghur, and 20.28% (95% CI: 18.72% to 21.84%) in Han. The age-standardized prevalence in male and female was 18.59% (95% CI: 16.09% to 21.10%) and 17.13% (95% CI: 15.13% to 19.13%) for Uyghur and 23.17% (95% CI: 20.60% to 25.74%) and 17.25% (95% CI: 15.50% to 19.01%) for Han. Hypertension prevalence stratified by ethnicity and sex and age was illustrated in Figure 1 and S-table 2. Uyghur female had a higher prevalence of overweight/obesity than Han female (P<0.001), but they had similar hypertension prevalence. Correspondingly, Uyghur male had similar overweight/obesity prevalence with Han male, but Han male had higher hypertension prevalence than that of Han (P<0.05) (Figure 1).

**Figure 2:**
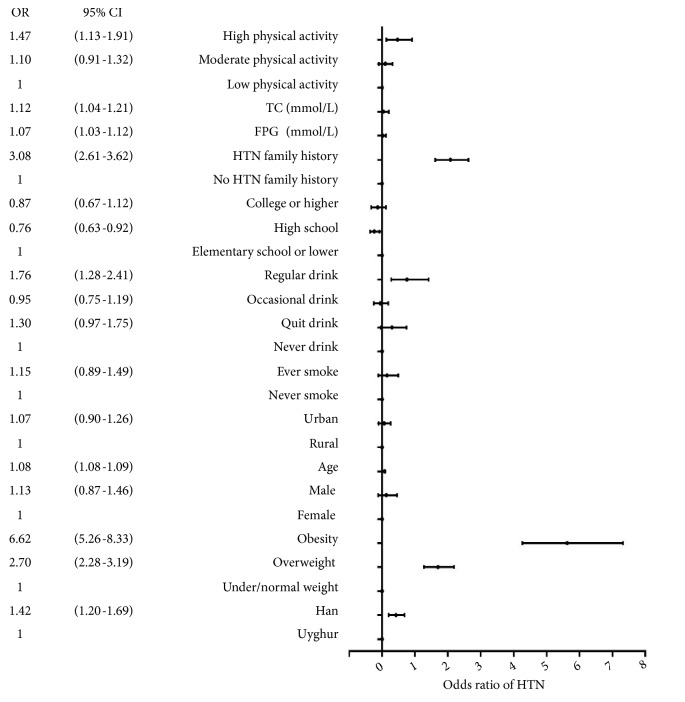
Associated factors of hypertension in participants. HTN: hypertension.

In the total population, overweight individuals had 2.70 times odds of hypertension compared with under/normal weight. For obese subjects, the OR was 6.62 (Table 2). Han adults had 1.42 (95% CI: 1.20 to 1.69) times odds than Uyghur of hypertension (Figure 2).

### 3.4. Optimal Cut-Off Values of Adiposity in Identifying Hypertension 

**Figure 3:**
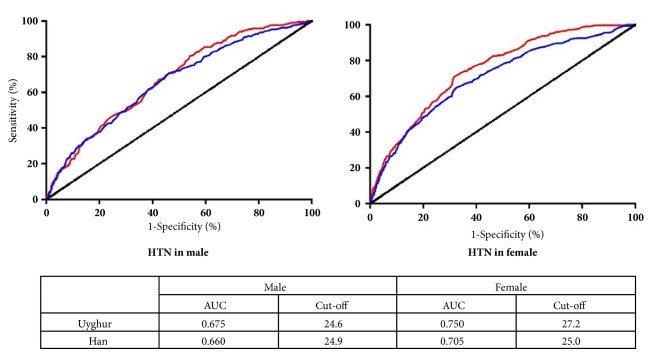
The ROC curves and optimal cut-off value for BMI to identify hypertension in both ethnic groups and sex. HTN: hypertension; AUC: area under the curve.

AUCs of BMI identifying hypertension were summarized in Figure 3, 0.662 (95% CI: 0.637-0.688) in men and 0.713 (95% CI: 0.693-0.734) in women. AUCs were found performed better in female than in male. In Uyghur, AUCs for male and female were 0.675 (95%CI: 0.637-0.713) and 0.750 (95% CI: 0.723-0.777); in Han, the AUCs for male and female were 0.660 (95% CI: 0.627-0.693) and 0.705 (95% CI: 0.675-0.734).

In male, the optimal cut-off values of adiposity identifying hypertension in Uyghur and Han were 24.6 kg/m^2^ and 24.9 kg/m^2^, respectively, but the correspond values in Uyghur and Han female were 27.2 kg/m^2^ and 25.0 kg/m^2^.

## 4. Discussion

This is the first population-based study that investigates the effect adiposity on hypertension in Uyghur. It sheds insights into a possible role of excess adiposity on blood pressure elevation among Uyghur Chinese, as well as the varied effect between different ethnic groups.

In both Uyghur and Han adults, the odds ratios of hypertension appeared stronger among individuals with higher adiposity. The sensitivity analysis, where BMI was replaced by body fat percentage, provided consistent result with present findings. Previous studies have observed ethnic disparities between Uyghur and other ethnic groups in chronic diseases prevalence, such as type 2 diabetes [11, 12, 20], dyslipidemia [21], and hypertension [13, 14]. Consistent with Gu's study [13], our study revealed that, compared with Han adults, Uyghur had lower prevalence of hypertension. By contrast, Tao's study [14] reported a higher prevalence in Uyghur than Han adults aged 35-74 years. The possible reason for this contradiction could be due to the heterogeneity of study population. For example, in Tao's study, they claimed that Uyghur Chinese were characterized by strong wine. However, Uyghur adults enrolled in our study had much lower rate of alcohol drinking than Han adults, which may be attributed to the alcohol abstinence.

Uyghur had generally high BMIs, which might be attributable to multiple factors, including lifestyle factors and economic developments. However, interestingly, in our study, Uyghur adults had lower hypertension prevalence than Han. It suggested that the impact of excess adiposity on blood pressure may be less strong in Uyghur than in Han adults. Like populations of Central Eurasia, Uyghur Chinese are genetically related to both Caucasian and East Asian populations [22, 23]. On the genetic level, ethnicity may be linked to the distribution of genetic polymorphisms related to obesity and hypertension [9, 24, 25]. For BMI, the absolute risk of hypertension tended to be higher among Asians compared with Caucasians [8, 10, 26]. The mixture of both Caucasian and Asian genetic background may partially explain the varied effect. Besides the genetic background difference, this varied effect could also be attributed to environmental factors. As the present study found out, Uyghur male had a much lower proportion of alcohol consumption than Han male. Among male never drinkers, the age-standardized prevalence of hypertension in Uyghur and Han declined to 15.07% and 16.93%, respectively. Additionally, in Uyghur and Han female, whose alcohol consumption was of no difference and in low level, the prevalence of hypertension was both low and similar (17.13% vs. 17.25%). Therefore, the difference of hypertension prevalence may be to some extent attributed to alcohol consumption.

Although heritability of BMI remained high over all age groups, the importance of environmental determinants for BMI increased with aging [27]. Therefore, to minimize the environmental influence, analyses conducted in relatively young individuals are recommended to know the relationship between body composition and hypertension [28]. The age- and sex-specific prevalence difference of hypertension between Uyghur and Han indicated that, in male obese participants, above 50 years old, the difference reduced as age increased (see S-table 2). The trend of prevalence difference suggested that Uyghur male may have accelerated vulnerability to hypertension with aging.

The adjusted means of DBP in Han were found higher than Uyghur adults. This difference was possibly attributable to genetic heterogeneity between Uyghur and Han. Since DBP was considered more strongly predicting cardiovascular disease risk in younger adulthood [1], hypertension-related health burden in Han maybe higher than Uyghur.

As AUCs of 0.6-0.7 are considered poor and 0.7-0.8 are fair [29], BMI performs better among female in predicting hypertension, which was consistent with Li's study [30] in which body fat percentage was used as index of body composition. This is probably because more complicated risk factors were clustered in male population, such as cigarette smoking and alcohol drinking, which could to some extent conceal the effect adiposity on blood pressure. The cut-off values of BMI in female revealed ethnicity disparities in adiposity effect on hypertension, but this effect was not observed in male participants.

This study adds evidence to previous observations indicating that excess adiposity has ethnic disparity in influencing hypertension. It implied that controlling body mass index to a normal range could have considerable potential for prevention of hypertension, which would lead to a broad public health implication. This study has several strengths. Firstly, by using a multistage stratified clustering sampling method, we selected representative samples of study population. Secondly, the strict quality control standards being implemented all through the survey guaranteed the data validity and reliability.

Limitations of this study should also be acknowledged: first, participants who were diagnosed with hypertension or other chronic diseases before the survey may have some lifestyle changes, thus leading to a biased estimation of the effect. Nonetheless, in the sensitivity analysis where individuals who had been diagnosed with hypertension before the survey were excluded, the ORs for BMI of hypertension enlarged. Likewise, among participants without diabetes, the ORs remain stable. Therefore, our study provides a conservative estimation of adiposity on hypertension. Second, central obesity was not measured, thus comparisons of associations among different body composition indices with hypertension were not available. But compared with BMI, WC were not found more strongly associated with prevalence of hypertension [10]. In this way, our finding provides credible evidence of the relationship between excess adiposity and hypertension. Thirdly, the sample size was insufficient when stratified analysis was performed, especially in young obese adults.

## Figures and Tables

**Table 1:** Demographic and clinical characteristics of Uyghur and Han adults of China, 2013.

	**Uyghur (n=2928)**				**Han (n=3097)**			
	**Male (n=1028)**		**Female (n=1900)**		**Male (n=1255)**		**Female (n=1842)**	
**Age, years (mean, SD)**	46.3	14.33	43.25^a^	11.69	47.71^d^	13.66	47.73^c^	12.45
**Educational attainment (n, %)**								
Illiterate	33	3.27	42	2.24	45	3.63^c^	195	10.66^ac^
Elementary school	215	21.31	411	21.94	135	10.89	274	14.97
Junior high school	241	23.89	469	25.04	386	31.13	502	27.43
Senior high school	241	23.89	420	22.42	289	23.31	378	20.66
College or higher	279	27.65	531	28.35	385	31.05	481	26.28
**Residential areas (n, %)**								
Urban	519	51.18	877	46.62^b^	800	64.41^c^	1150	62.77^c^
Rural	495	48.82	1004	53.38	442	35.59	682	37.23
**BMI, kg/m** ^**2**^ ** (n, %)**								
<25	454	44.42	841	44.29^a^	612	48.84^d^	1122	60.91^ac^
≥25	457	44.72	706	37.18	531	42.38	581	31.54
≥30	111	10.86	352	18.54	110	8.78	139	7.55
**Cigarette smoking (n, %)**								
Ever smoking	711	70.19	3	0.16^a^	906	73.01	34	1.85^ac^
Never smoking	302	29.81	1881	99.84	335	26.99	1799	98.15
**Alcohol drink (n, %)**								
Quit drink	252	25.07	4	0.21^a^	137	11.16^c^	36	1.97^ac^
Regular drink	71	7.06	1	0.05	295	24.02	33	1.81
Occasional drink	267	26.57	7	0.37	560	45.60	413	22.64
Never drink	415	41.29	1873	99.36	236	19.22	1342	73.57
**Physical activity (n, %)**								
Low	174	17.58	620	34.75^a^	166	13.46^c^	401	22.09^ac^
Moderate	648	65.45	1002	56.17	825	66.91	1218	67.11
High	168	16.97	162	9.08	242	19.63	196	10.80
**FPG, mmol/L (mean, SD)**	5.30	1.72	5.12^a^	1.48	5.30	1.57	5.20	1.38
**TC, mmol/L (mean, SD)**	4.87	1.08	4.76^b^	1.02	4.69^c^	0.94	4.81^a^	1.00
**TG, mmol/L (mean, SD)** ^**∗**^	2.03	2.05	1.4^a^	1.06	2.02	2.12	1.49^ad^	1.23
**LDL-C, mmol/L (mean, SD)**	2.98	0.79	2.89^a^	0.82	2.75^c^	0.74	2.77^c^	0.78
**HDL-C, mmol/L (mean, SD)**	1.26	0.25	1.42^a^	0.26	1.34^c^	0.28	1.52^ac^	0.30
**SBP, mmHg (mean, SD)**	123.33	15.71	117.06^a^	18.29	124.30	14.92	117.22^a^	17.06
**DBP, mmHg (mean, SD)**	73.89	11.16	72.12	11.80	76.84^c^	11.04	71.95^a^	10.35
**Hypertension (n, %)**	219	21.53	321	17.05^a^	344	27.79^c^	388	21.19^ac^

Values are presented as mean and SD for continuous data or number and percentage (%) for categorized data.

a: comparison between male and female in the same ethnic group, P<0.01; b: comparison between male and female in the same ethnic group, P<0.05; c: comparison between Uyghur and Han in the same sex group, P<0.01; d: comparison between Uyghur and Han in the same sex group, P<0.05. Abbreviations: BMI, body mass index (calculated as weight in kilograms divided by height in meters squared.); FPG: fasting plasma glucose; SBP: systolic blood pressure; DBP: diastolic blood pressure; TC, triglyceride; TC, total cholesterol, HDL-C, high density lipoprotein cholesterol; and LDL-C: low-density lipoprotein cholesterol.

**Table 2:** Logistic regression models to estimate the odds ratios between BMI and hypertension in Uyghur and Han adults in China, 2013^*∗*^ .

	**Model 1**						**Model 2**					
	Overweight^#^			Obesity^#^			Overweight^#^			Obesity^#^		
	OR	95% CI		OR	95% CI		OR	95% CI		OR	95% CI	
**Overall**	2.65	2.27	3.10	6.15	5.02	7.53	2.70	2.28	3.19	6.62	5.26	8.33
** Uyghur**	2.88	2.23	3.71	6.56	4.88	8.83	2.67	2.00	3.57	6.04	4.29	8.51
** Han**	2.66	2.18	3.25	7.05	5.18	9.61	2.74	2.20	3.40	7.58	5.37	10.69
**Male **	2.60	2.06	3.27	6.24	4.43	8.77	2.42	1.87	3.11	6.23	4.25	9.14
** Uyghur**	2.71	1.87	3.94	5.54	3.31	9.27	2.36	1.54	3.62	4.64	2.58	8.33
** Han **	2.63	1.96	3.53	7.65	4.79	12.23	2.50	1.81	3.45	8.49	4.98	14.46
**Female **	2.59	2.10	3.20	5.92	4.58	7.65	2.68	2.12	3.39	6.03	4.47	8.14
** Uyghur**	2.93	2.06	4.16	6.95	4.78	10.11	2.78	1.86	4.15	6.87	4.42	10.69
** Han **	2.54	1.93	3.35	5.95	3.92	9.05	2.77	2.05	3.72	6.71	4.24	10.63

^*∗*^In Model 1, only age and sex were adjusted. In Model 2, covariates were selected differently based on varied logistic regression models. For the overall model, ethnicity, sex, age, residential areas, educational attainment, family history of hypertension, smoking status, alcohol drinking, physical activity, FPG and TC were adjusted. For male and female adults, covariates were included separately in the model except sex. The ethnic- and sex-specific logistic model included covariates except ethnicity and sex. For female participants, smoking status and alcohol drinking were not included as covariates because of the limited number. Age was set as continuous covariate, and other variables were set as indicators.

^#^reference group=under/normal group.

## Data Availability

An application for the original data can be provided via http://www.bmicc.cn/web/share/home.

## Supplementary Materials

Supplementary Materials S-table 1 describes the association between body mass index (BMI) and blood pressure (both SBP and DBP) in Uyghur and Han adults, stratified by ethnic groups and sex. S-table 2 presents the sex- and age-specific prevalence of hypertension among Uyghur and Han adults. S-table 3 presents the Area under the ROC curve (AUC) of BMI for identifying hypertension in Uyghur and Han adults, stratified by sex and age groups. S-figure 1 shows the scatter plots and linear regression fit for BMI and SBP, with 95% confidence interval and 95% value range.
