# Supplementary material for: Effect of BMI and Its Optimal Cut-Off Value in Identifying Hypertension in Uyghur and Han Chinese: A Biethnic Study from the China National Health Survey (CNHS)
Source: Int J Hypertens. 2018 Dec 11;2018:1508083. doi: 10.1155/2018/1508083 (PMC6311287; doi:10.1155/2018/1508083)
Supplement: Supplementary Materials — S-table 1 describes the association between body mass index (BMI) and blood pressure (both SBP and DBP) in Uyghur and Han adults, stratified by ethnic groups and sex. S-table 2 presents the sex- and age-specific prevalence of hypertension among Uyghur and Han adults. S-table 3 presents the Area under the ROC curve (AUC) of BMI for identifying hypertension in Uyghur and Han adults, stratified by sex and age groups. S-figure 1 shows the scatter plots and linear regression fit for BMI and SBP, with 95% confidence interval and 95% value range. [file 1508083.f1.docx]

Supplementary materials

S-table 1 describes the association between body mass index (BMI) and blood pressure (both SBP and DBP) in Uyghur and Han adults, stratified by ethnic groups and sex.

S-table 1 Associations between BMI and blood pressure stratified by ethnic groups and sex.

|  | Male | | | | | | Female | | | | | | |
| --- | --- | --- | --- | --- | --- | --- | --- | --- | --- | --- | --- | --- | --- |
|  | BMI (kg/m^2^) | | | | | | BMI (kg/m^2^) | | | | | | |
| UYGHUR | **<25** | | **25-** | | **30-** | |  | **<25** | | **25-** | | **30-** | |
| SBP (mmHg)^*^ | 118.30 | 14.52 | 126.27 | 15.45 | 131.81 | 14.88 |  | 109.72 | 15.05 | 120.16 | 17.31 | 128.28 | 19.72 |
| DBP (mmHg)^*^ | 69.97 | 10.36 | 76.02 | 10.56 | 81.13 | 10.87 |  | 67.07 | 9.95 | 74.12 | 10.94 | 80.10 | 11.92 |
| Blood pressure (SBP/DBP) ^#^ | | |  |  |  |  |  |  |  |  |  |  |  |
| <120 and <80 | 269 | 59.25 | 161 | 35.23 | 24 | 21.62 |  | 681 | 80.98 | 383 | 54.25 | 123 | 34.94 |
| 120- or 80- | 143 | 31.50 | 212 | 46.39 | 53 | 47.75 |  | 117 | 13.91 | 222 | 31.44 | 136 | 38.64 |
| 140- or 90- | 33 | 7.27 | 62 | 13.57 | 23 | 20.72 |  | 30 | 3.57 | 72 | 10.20 | 53 | 15.06 |
| ≥160 or ≥100 | 9 | 1.98 | 22 | 4.81 | 11 | 9.91 |  | 13 | 1.55 | 29 | 4.11 | 40 | 11.36 |
| HAN |  |  |  |  |  |  |  |  |  |  |  |  |  |
| SBP (mmHg)^*^ | 119.87 | 14.22 | 127.14 | 13.92 | 135.44 | 14.44 |  | 112.79 | 15.56 | 122.44 | 16.65 | 130.93 | 16.86 |
| DBP (mmHg)^*^ | 73.63 | 10.06 | 78.67 | 10.41 | 86.06 | 12.1 |  | 69.35 | 9.37 | 74.83 | 10.1 | 80.74 | 10.86 |
| Blood pressure (SBP/DBP) ^#^ | | | |  |  |  |  |  |  |  |  |  |  |
| <120 and <80 | 324 | 52.94 | 155 | 29.19 | 11 | 10.00 |  | 800 | 71.30 | 270 | 46.47 | 35 | 25.18 |
| 120- or 80- | 216 | 35.29 | 263 | 49.53 | 56 | 50.91 |  | 241 | 21.48 | 218 | 37.52 | 59 | 42.45 |
| 140- or 90- | 60 | 9.80 | 89 | 16.76 | 26 | 23.64 |  | 62 | 5.53 | 68 | 11.70 | 32 | 23.02 |
| ≥160 or ≥100 | 12 | 1.96 | 24 | 4.52 | 17 | 15.45 |  | 19 | 1.69 | 25 | 4.30 | 13 | 9.35 |

^*^both in male and female, P<0.0001, P<0.0001 for all subgroups two-two comparisons.

^#^both in male and female, P <0.0001;

S-table 2 presents the sex- and age-specific prevalence of hypertension among Uyghur and Han adults.

S-table 2 Hypertension prevalence among Uyghur and Han adults, stratified by sex and age-groups (n, %)

|  | Uyghur | | | | | | Han | | | | | |
| --- | --- | --- | --- | --- | --- | --- | --- | --- | --- | --- | --- | --- |
|  | BMI (kg/m^2^) | | | | | | BMI (kg/m^2^) | | | | | |
|  | **<25** | | **25-** | | **30-** | | **<25** | | **25-** | | **30-** | |
|  | n | % | n | % | n | % | n | % | n | % | n | % |
| Male |  |  |  |  |  |  |  |  |  |  |  |  |
| 20- | 4 | 3.54 | 3 | 8.33 | 1 | 11.11 | 1 | 1.30 | 5 | 12.20 | 1 | 11.11 |
| 30- | 3 | 4.35 | 4 | 4.44 | 6 | 25.00 | 6 | 6.32 | 14 | 16.87 | 12 | 41.38 |
| 40- | 6 | 6.90 | 30 | 20.83 | 8 | 25.00 | 22 | 13.66 | 45 | 26.95 | 16 | 50.00 |
| 50- | 16 | 20.00 | 39 | 38.61 | 17 | 62.96 | 34 | 24.82 | 60 | 45.45 | 19 | 76.00 |
| 60- | 27 | 26.47 | 44 | 51.76 | 11 | 61.11 | 46 | 33.82 | 54 | 53.47 | 9 | 69.23 |
| Total | 56 | 12.42 | 120 | 26.32 | 43 | 39.09 | 109 | 17.99 | 178 | 33.97 | 57 | 52.78 |
| Female |  |  |  |  |  |  |  |  |  |  |  |  |
| 20- | 1 | 0.48 | 1 | 2.44 | 0 | 0 | 0 | 0 | 1 | 6.67 | 0 | 0 |
| 30- | 5 | 2.06 | 8 | 5.37 | 12 | 18.75 | 9 | 3.61 | 8 | 10.67 | 6 | 37.50 |
| 40- | 14 | 7.00 | 45 | 15.73 | 46 | 29.87 | 27 | 6.96 | 32 | 16.49 | 10 | 27.78 |
| 50- | 17 | 16.50 | 55 | 34.38 | 41 | 51.25 | 41 | 18.89 | 54 | 35.76 | 18 | 51.43 |
| 60- | 17 | 22.67 | 29 | 43.94 | 30 | 69.77 | 60 | 39.47 | 85 | 58.62 | 37 | 77.08 |
| Total | 54 | 6.50 | 138 | 19.66 | 129 | 36.86 | 137 | 12.32 | 180 | 31.03 | 71 | 51.08 |

*in the same age-group, P for trend<0.05; #in the same age-group, P for trend <0.0001

^&^in the same BMI category, P for trend<0.01; ^Ψ^ in the same BMI category, P for trend<0.001

S-table 3 presents the Area under the ROC curve (AUC) of BMI for identifying hypertension in Uyghur and Han adults, stratified by sex and age groups.

S-table 3 AUC of BMI for identifying hypertension.

|  | Uyghur | | | | | Han | | | | | Overall | | | | |
| --- | --- | --- | --- | --- | --- | --- | --- | --- | --- | --- | --- | --- | --- | --- | --- |
| *Hypertension* | n | % | AUC | 95%CI | | n | % | AUC | 95%CI | | N | % | AUC | 95%CI | |
| Male | 219 | 21.5 | **0.675** | 0.637 | 0.713 | 344 | 27.8 | **0.660** | 0.627 | 0.693 | 563 | 25.0 | **0.662** | 0.637 | 0.688 |
| 20- | 8 | 5.06 | 0.732 | 0.602 | 0.861 | 7 | 5.51 | 0.744 | 0.513 | 0.974 | 15 | 5.26 | 0.729 | 0.603 | 0.855 |
| 30- | 13 | 7.10 | 0.660 | 0.482 | 0.838 | 32 | 15.5 | 0.764 | 0.677 | 0.851 | 45 | 11.5 | 0.720 | 0.640 | 0.801 |
| 40- | 44 | 16.7 | 0.638 | 0.557 | 0.718 | 83 | 23.1 | 0.689 | 0.623 | 0.756 | 127 | 20.4 | 0.667 | 0.615 | 0.718 |
| 50- | 72 | 34.6 | 0.722 | 0.651 | 0.793 | 113 | 38.4 | 0.673 | 0.611 | 0.736 | 185 | 36.9 | 0.688 | 0.641 | 0.735 |
| 60-80 | 82 | 40.0 | 0.693 | 0.621 | 0.766 | 109 | 43.6 | 0.616 | 0.545 | 0.686 | 191 | 42.0 | 0.650 | 0.599 | 0.701 |
| Female | 321 | 17.1 | **0.750** | 0.723 | 0.777 | 388 | 21.2 | **0.705** | 0.675 | 0.734 | 709 | 19.1 | **0.713** | 0.693 | 0.734 |
| 20- | 2 | 0.770 | 0.830 | 0.591 | 1.000 | 1 | 0.800 | 0.960 | 0.925 | 0.994 | 3 | 0.780 | 0.878 | 0.740 | 1.000 |
| 30- | 25 | 5.48 | 0.778 | 0.689 | 0.867 | 23 | 6.76 | 0.728 | 0.608 | 0.847 | 48 | 6.03 | 0.747 | 0.673 | 0.821 |
| 40- | 105 | 16.4 | 0.712 | 0.659 | 0.764 | 69 | 11.2 | 0.660 | 0.589 | 0.732 | 174 | 13.8 | 0.700 | 0.657 | 0.742 |
| 50- | 113 | 32.9 | 0.675 | 0.616 | 0.734 | 113 | 28.0 | 0.655 | 0.594 | 0.715 | 226 | 30.3 | 0.669 | 0.627 | 0.711 |
| 60-80 | 76 | 41.3 | 0.738 | 0.667 | 0.810 | 182 | 52.8 | 0.659 | 0.602 | 0.716 | 258 | 48.8 | 0.679 | 0.633 | 0.724 |

Supplementary figure

S-figure 1 shows the scatter plots and linear regression fit for BMI and SBP, with 95% confidence interval and 95% value range.


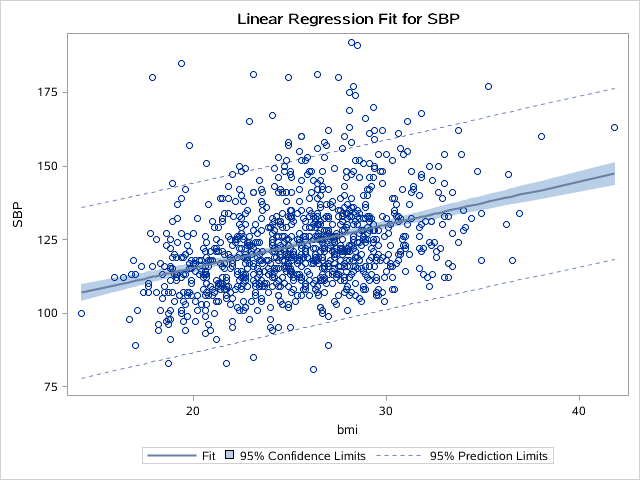

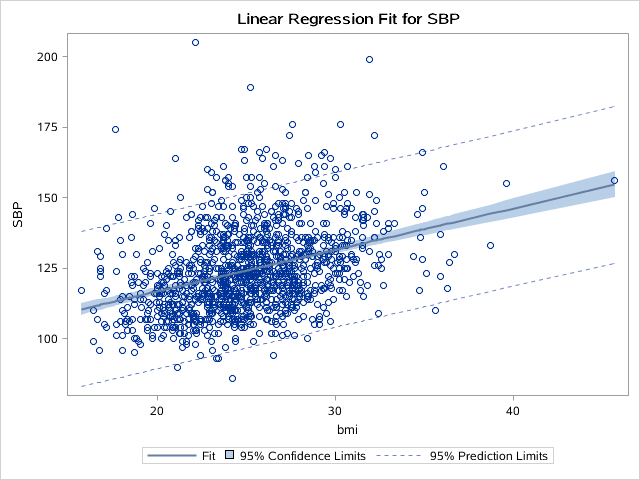

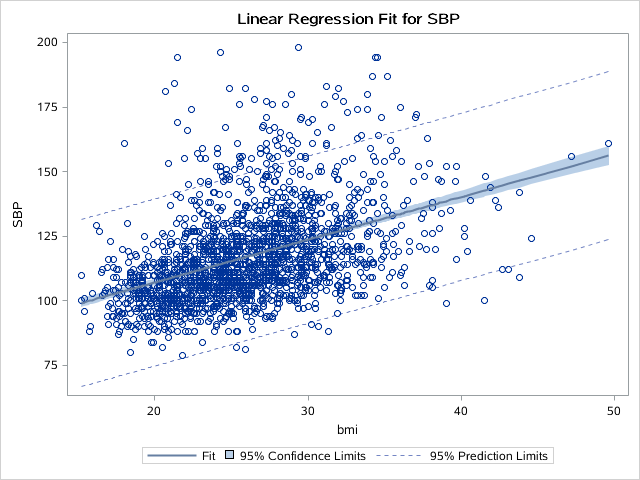

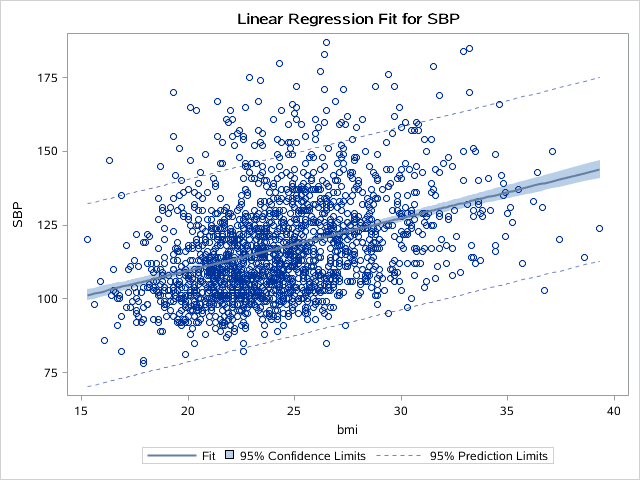


S-figure 1 linear regression fit for SBP and BMI, stratified by ethnic group and sex

A: Uyghur male

B: Han male

C: Uyghur female

D: Han female
